# The central role of housing key workers in supporting healthcare interactions for people experiencing homelessness and implications for palliative care: a qualitative study

**DOI:** 10.1186/s12904-024-01598-x

**Published:** 2024-12-02

**Authors:** Merryn Gott, Lisa Williams, Janine Wiles, Stella Black, Tess Moeke-Maxwell, Jackie Robinson, Te Ārai Palliative Care and End of Life Research Group

**Affiliations:** 1 Te Kura Tapuhi/The School of Nursing, Waipapa Taumata Rau/The University of Auckland, 85 Park Road, Auckland, Aotearoa New Zealand; 2 The School of Population Health, Waipapa Taumata Rau/The University of Auckland, Auckland, Aotearoa New Zealand; 3 https://www.tearairesearchgroup.net

**Keywords:** Palliative care, Homelessness, Housing key worker, Healthcare, Hospital, Emergency department, Equity

## Abstract

**Background:**

People experiencing homelessness access specialist palliative care late in their illness trajectory, if at all. There is also little evidence they receive generalist palliative care or are given opportunities to engage in Advance Care Planning. This qualitative study describes the central role of key workers in supporting access to healthcare in homeless communities and identifies implications for improving palliative care provision.

**Methods:**

Qualitative data were collected via focus groups and individual interviews with staff working for a key provider of support and housing/accommodation for people experiencing homelessness in an urban area of Aotearoa New Zealand.

**Results:**

The ability to provide palliative care for people experiencing homelessness is dependent upon supporting engagement with mainstream health services. It is here that we identified the key worker role as central due to the complex and expert work they undertake to facilitate healthcare access for their clients. As a result of the high burden of chronic conditions this community experiences, most of this work related to support managing serious conditions, as well as death and dying. Key workers often went ‘above and beyond’ to support their clients in engaging with mainstream health services, during outpatient appointments, hospital admissions and in emergency department settings. They felt clinicians in these settings did not recognise the knowledge they held about the person, or their skills in terms of providing trauma informed care. The inflexibility of current care provision, as well as people experiencing homelessness feeling stigmatised, and neither valued nor respected in these settings, also created barriers to receiving care.

**Conclusions:**

New models of palliative care are required which recognise the central role of non-health care key support staff and engage them more actively in supporting people experiencing homelessness when they interact with mainstream health services. Such models will need to be responsive to the nature and complexity of palliative care need in this population and facilitate support for people who typically do not see healthcare spaces as safe. The trusted relationships key workers have developed over time are crucial resources for identifying palliative care need and supporting access to palliative care for people experiencing homelessness.

**Supplementary Information:**

The online version contains supplementary material available at 10.1186/s12904-024-01598-x.

## Background

The ‘equity turn’ [1] in palliative care is being driven by increasing evidence of large disparities in end-of-life experience both within, and between, countries [2]. Rooted in political, social and economic structures, such disparities manifest themselves in differential access to specialist palliative care services according to an individual’s social position(s). For those who experience structural disadvantage, end of life experience can also be marred by stigma and discrimination when accessing mainstream health services [3–5]. There is evidence that this is certainly the case for people who are homeless, or precariously or vulnerably housed: for example, those living in cars or ‘couch surfing’ with family and friends, or even those moving in and out of transitory supported housing. Indeed, whilst ‘home’ is central to contemporary palliative care policy [6], the unwritten assumption is that ‘home’ is a comfortable warm house with sufficient food in the fridge and family who are able to provide care [7].

People experiencing homelessness are likely to endure more complex and significant palliative care needs than the housed population due to accelerated ageing, high frequency of untreated chronic conditions, histories of trauma, and experiences of addiction which complicate pain management [8]. An ethnographic study by Stajduhar et al. [8] concluded that, for these reasons, all people experiencing homelessness are at risk of dying at any time. It is therefore worrying that existing international evidence confirms that homeless and vulnerably housed people experience numerous barriers to accessing both healthcare and specialist palliative care services [9]. These relate not just to the physical accessibility of services, but also to their suitability. Institutional spaces such as hospitals are often perceived and experienced as unsafe sites of discrimination and previous trauma [10], particularly for Indigenous people [11]. People experiencing substance use disorders may be labeled as drug seekers and denied pain medication [12], as well as be stigmatised and denied the ability to use substances at the end of life when this is desired [13]. A focus on day-to-day survival can also override physical health concerns [8]. As a result, people experiencing homelessness typically access specialist palliative care very late in their illness trajectory, if at all [14, 15]. There is also little evidence that they receive generalist palliative care, are given opportunities to engage in planning for the end-of-life, or have the ability to choose the places in which they die [11, 16]. Moreover, palliative care as it is currently provided may not be an attractive proposition for homeless communities [14, 17].

These findings are contrary to World Health Organization recommendations to introduce a palliative approach to care early in the course of all life-limiting illness [18]. They also point to a need to better understand interactions between people experiencing homelessness and mainstream healthcare services, to identify opportunities to introduce a palliative approach to care or initiate a referral to specialist palliative care. Whilst models of palliative care for homeless and vulnerably housed people remain in their infancy, there is evidence pointing to the important role played by non-health care teams who support this community [15, 19–21]. This includes social care professionals, typically with no health-related education or training, whose role it is to establish relationships and identify potential pathways to housing and other support for individuals experiencing homelessness. Whilst a range of terminology is used to describe these workers both nationally and internationally, for the purposes of this study we will refer to them as housing key workers.

### Aim

To explore the role of housing key workers in supporting access to health services by homeless and vulnerably housed people and identify implications for palliative care.

## Methodology

### Critical framework

The critical framework for this research relies on social constructionism. Rather than asserting knowledge is the product of objective, unbiased observations of phenomena, we contend that it is historically and culturally specific [22]. We draw from critical social theory and Indigenous and feminist approaches to recognise the roles ideology and history play in concealing the ways social structures oppress and control people, particularly those with marginalised social identities, such as those experiencing homelessness. We also acknowledge that all people have multiple, intersecting social identities and looked for how these intersections operated within our analysis.

### Study setting and ethical issues

This study was set in a large urban area in Aotearoa New Zealand and undertaken in partnership with a provider of services to homeless and vulnerably housed people. This paper addresses a key aim of the study, namely to understand the circumstances and situations in which their clients – who they refer to as street whānau (whānau is a te reo Māori language term for family group) – are experiencing serious illness and dying, and the work undertaken by their key workers in this space. Of note, Aotearoa New Zealand has responsibilities for improving health outcomes for Māori enshrined in Te Tiriti o Waitangi (The Treaty of Waitangi) and contemporary health policy. As a result of colonisation and racism Māori are over-represented within the homeless population [23] and experience considerable challenges in accessing culturally safe and timely healthcare, including palliative care [24, 25]. Te Ārai Palliative Care and End of Life Research Group work with the support of Te Ārai Kāhui Kaumātua, a group of Māori elder cultural advisors to ensure culturally safe research practices with Māori participants. Māori participants were offered the option of being interviewed by a Māori researcher (TMM). Where this was preferred, Māori participants were given the opportunity for karakia (prayers, incantations, chants) to be said before the interview; pepeha (introductions) were also shared as part of establishing whakawhanaungatanga (relationship connections).

### Methods

Data were collected through four in-person focus groups containing either 3 or 4 participants and six semi-structured interviews. Of the interviews, one was conducted in-person on-site at the organisation and five were conducted via zoom. Focus groups were used because they have been found to be helpful when working with teams in which people are known to each other [26] and allow for the expression of diverse viewpoints about a shared topic of interest [27]. Semi-structured interviews were used where people were unable to attend a focus group and for Māori participants who preferred an individual interview with a Māori researcher. The same topic guide was used for the focus groups and the interviews, with flexibility also provided to follow up issues of interest. These were developed by JR following an exploration of the relevant literature and reviewed and revised by the research team (Appendix 1). The duration of the focus groups was between 1 and 1.5 h. The interviews lasted between 45 min and 1 h. All focus groups and interviews were audio-recorded with participant consent and refreshments were provided.

The study received ethics approval from the Health and Disabilities Ethics Committees, reference number HRC#9613.

### Participants

The participants were mainly non-healthcare staff, employed to support street whānau and people living in transitional and emergency housing. See Table 1 for a breakdown of selected demographics.

**Table 1:** Participant demographics

**Roles**		**Time in profession or industry**	
Key Worker	12	Less than 1 year	1
Social worker	1	Between 1—2 years	3
Health & Social Services Coordinator	1	Between 2—3 years	3
Nurse	3	Between 3—4 years	1
Residential Services Manager	1	Between 6 -7 years	2
Doctor	2	Between 8—10 years	2
		18 years	1
**TOTAL**	**20**	22 years	1
		Undisclosed	6
**Gender**			
Women	17		
Men	3	**Age**	
		20's	2
		30's	1
		40's	3
		50's	3
		60's	3
		70's	1
		Undisclosed	7

### Analysis

Over the period of six months, the focus groups and interview transcripts were closely read by members of the research team (MG, JW, JR, LW) in bi-monthly meetings. The goal of these meetings was to identify and discuss key ideas and categories and thereby develop a coding framework. During this process, the team examined the role of housing key workers as it became apparent that their activities were central to healthcare access by street whānau and thus have important implications for palliative care. Subseqently, only data related to the role of key workers was used in the analysis for this paper. Of note, this role involves establishing relationships and identifying pathways to housing for individuals and whānau who are experiencing homelessness. People come to this role with diverse skills in social services and housing.

During discussions, the research team identified resonances with the *Knowing, Doing and Negotiating Framework* developed by JW, MG, TMM, and LW during a previous study involving family and whānau members who provided unpaid care to support people in advanced age at the end of life [28]. ‘Knowing’ underpins the doing and negotiating aspects of care and support. It involves understanding what, when, and how a task needs to be done. It also includes an awareness of available resources and services (or the lack of them) as well as the preferences and context of the person they are supporting. ‘Doing’ comprises the specific tasks involved in supporting someone. They include personal, physical and practical tasks expressed in such processes as mediating and advocating, information gathering, supporting decision-making, accompanying people to appointments, managing financial tasks, household work, personal care, and emotional support. Finally, ‘negotiating’ refers to the ongoing skill needed to balance the diverse, and often conflicting, aspects of providing support. Negotiating may be intra-personal, with the caregiver deciding within themselves how much they can physically or emotionally handle, or it may be an interpersonal interaction involving interactions with others, including the person receiving care, and other paid and unpaid supporters and other members of networks.

MG re-read all the focus group and interview data in line with the Knowing, Doing and Negotiating framework, and undertook additional preliminary coding that centred on housing key worker tasks, healthcare access, resourcing, and post-death in relation to the study research question. She drew on the seminal ideas and categories developed during preliminary meetings to draft a story that synthesised the identified themes in the data, related these to the framework, and evidenced them with supporting quotations, a process akin to storyline in grounded theory research [29]. MG and LW then refined these themes and LW subsequently analysed all transcripts in accordance with this new framework.

Most of this storyline related to the large amount of data gathered about mainstream healthcare services; little was shared by participants about access to hospice and specialist palliative care as most deaths were described as occurring suddenly meaning there wasn’t seen to be time to initiate a referral. We identified interactions with mainstream healthcare services as providing an opportunity for both generalist palliative care provision, as well as referral to specialist palliative care. We also were attentive to, and coded for, barriers and facilitators to housing key workers carrying out their knowing, doing and negotiating work. Throughout this process, the researchers openly discussed with each other our assumptions and reactions to the data as a means of engaging reflexively with the analysis.

## Findings

### One of your whānau members: housing key workers as de facto family

To grasp the significance of housing key workers, it is necessary to understand how they filled gaps in the support available to street whānau, most of whom did not have regular contact with their family of origin. Moreover, whilst participants described “great connections” amongst many street whānau, their histories of trauma and negative experiences with mainstream health services compromised their ability to provide support for each other in the case of serious illness and dying:

*They’ve more often than not burned their bridges with their families for whatever reason or they’ve walked away from the family because of trauma. So there’s quite often a total disconnect from blood family, so then Streeties become the whāngai [adopted] family, but they’re all traumatised, they’re all trying to re-parent each other.* (FG 3)

Participants described a high rate of death and dying within their homeless communities. This meant that serious illness did not always trigger the same response as it would in the housed population within their immediate social network:

*I think lots of them just accept or have it in their heart that, like, you know, we’re Streeties, this is what happens. It’s like a norm for them, so if they know that that kind of stuff is happening – like, us, if we’d heard that about our close friends we’d be like, ‘oh no, we’ve got to go see that person straightaway’. Whereas with them I really feel the mentality is like, ‘another one’.* (FG 3)

This reaction by street whānau is likely rooted in their often multiple and lifetime experiences of trauma. These experiences meant that loss and grief, whilst felt deeply, may not always be articulated or openly demonstrated. Participants did describe street whānau acting as caregivers to support each other, and gave examples of practical and emotional acts of support such as checking in on each other and sharing food, clothing, and other resources. However, housing key workers provided the majority of support in accessing healthcare, which they achieved by centering trusting relationships in a similar way as they would with family and whānau relationships. Participants frequently reflected on the same values they used in their personal relationships to underpin their relationships with their street whānau, emphasizing how they would continue to be there no matter how challenging the situation. As one housing key worker put it when responding to a question about how they advocate for street whānau to engage with services:

*It’s so huge to gain that trust and earn that trust … I’ve always had in my head, ‘treat those people with dignity and respect and like it’s one of your whānau members’. (FG1)*

Others in the group agreed about the importance of empathy, connection, and trust in achieving this.

We identified two important principles or professional tenets underpinning all support provided by housing key workers. The first relates to self-determination, autonomy, and choice for street whānau. As one participant shared:

*We remind ourselves that they are the experts of their own lives and we’re just here to provide the options. But it’s up to them, you know, it’s up to them to make their own decisions just as we all make our own decisions.* (Int-1)

This core principle was consistently demonstrated as participants described a wide variety of interactions with street whānau and healthcare services, particularly in highly charged or intensely emotional situations. Secondly, participants emphasized the value of working hard at establishing a trusted, familial-type relationship as an ideal. These principles weaved through all aspects of their knowing, doing and negotiating work, which we elaborate on in more detail below.

### So many barriers, so much distrust: barriers to healthcare access for street whānau

The work undertaken by housing key workers in relation to healthcare access must be understood within the context of the significant barriers that inhibit street whānau from seeking help for a health problem. Participants described the low priority whānau themselves give to their physical health as well as the competing demands which limit the time available for them to attend healthcare appointments. As one housing key worker described:

*Several times I've had people say to me, ‘look, right now I might be [acutely unwell] but I won't let you send me to the hospital because if I don’t go to x appointment, or if I don’t go to this appointment with the foodbank I'm not gonna have food in my fridge to go home to. There’s no point in me living if I don’t have food in my fridge.’ And we’ve had – going alongside that. . . 'cause I owe Fred money, Fred wants his money by 12 o'clock today’. So that is their main thing, I can’t go to the appointment, I owe Fred the money, Fred’s got to be paid today. So the appointment, totally ignored. (FG5)*

Participants described how serious mental health problems, as well as alcohol and substance use and the accompanying impairment they caused, impacted on street whānau willingness to engage with health services. Similarly, they identified previous negative and traumatic experiences with mainstream health services, and institutions in general, as a key factor in whānau resistance to entering an institution such as a public hospital. This resistance was so strong that participants discussed occasions when street whānau had not wanted to be in an institution, even if they knew that not seeking healthcare would result in their death. As one participant shared in a focus group during a discussion about how street whānau are reluctant to engage with institutions because of poor past experiences and broken trust:

They just don’t feel that what they wish for their treatment plan is being honoured or respected. [My whānau clients] are coming from experiences of institutions, so, you know, so they would prefer to actually just go under the bridge and pass away there than having to be locked up.

And also with that, because they feel nobody cares anyway.

Yeah.

[they feel that] ‘if I go and pass away, nobody’s going to care.’

(FG-4)

The inflexibility of the healthcare system also inhibited access to healthcare. Participants discussed how healthcare is set up for a ‘straightforward, middle-class person’ rather than homeless or vulnerably housed people. Something as ubiquitous (for the middle class) as a mobile phone affected street whānau ability to make appointments and keep updated:

*A lot of our people don’t hold onto their phones, … they don’t take calls from unknown numbers, so there’s lots of barriers just in contacting people and having credit on [their] phone to have to ring people back. Yeah, there’s so many – what works for a very middle class straightforward person that are reliable and will return a phone call and want to have their appointment and can manage to get there, they can drive themselves there or they can get a family member to – all those things are not possible in this cohort.*

(FG-4)

The well-established difficulties for Māori and Pacific people in accessing, and receiving high-quality safe care, from a western health service, and the additional overlay of the ongoing trauma of colonization, was also discussed. As one participant stated:

*A large proportion of the population that we’re supporting is Māori and/or Pacifika. You know it might, to us it might seem quite plain and simple to access healthcare, but for them there’s so many barriers. And so much distrust within the healthcare system, that... they'd rather just avoid it until it becomes so detrimental that they're in a crisis*. (Int-1)

When these factors are considered together, it is understandable why key workers described a resistance from most street whānau to access healthcare and subsequently remain engaged with healthcare services. Whilst participants were explicit that they were only sharing their perspective and not that of their street whānau, our analysis confirmed that they undertook a substantive amount of work related to healthcare access, ranging from decision-making through to attendance at a health appointment or a hospitalization.

### Small deposits in the trust bank: the role of housing key workers in supporting the decision to seek treatment for a health problem

Supporting healthcare access for street whānau began with getting to *know* them and building a trusting relationship over time. For example, in a discussion about how they support street whānau to access healthcare services, one participant noted:

*Honestly, the work is done before you get to that point. Everything that we can do is not really done there, it’s done prior to, so it’s building trust, building relationships, showing up when you say you’re gonna show up, doing things. Even when they’re pushing you away, showing that look, [my response is] ‘okay, that’s fine, like I know you were abusive yesterday but I’ve still completed this thing for you and you don’t have to do anything for me in return. You know, it’s just here if you need it’. Like, small deposits in the trust bank.* (FG-3)

In addition, housing key workers had to undertake a lot of *negotiation* work to persuade street whānau to access healthcare. Examples provided included inviting street whānau to meet over a meal at the organisation’s onsite dining room and, once they got used to that, introducing the fact that there was a primary care clinic on site where they could get help. Other strategies included bringing a nurse to meet them, finding them and driving them to appointments, and attending appointments with them. This could be particularly important during tele-health appointments where they didn’t have access to the technology to facilitate their attendance and/or understand how to use it. In addition to having a trusted relationship, a flexible and supportive organisational culture on the part of the employing organisation was identified as critical, especially in crisis situations:

They said he had to be in hospital now and so, yeah, I just made a big mission. But he didn’t really care, you know, when like I was going oh, when I got him, I said, ‘I’m gonna hold you, I’m gonna take you –’

Int: Why do you think he didn’t care or didn’t seem to care?

He didn’t, no, he said, ‘no, I’ll be alright’. And I said, ‘no, do you know that they’ve been saying that you’ve got blah, blah, blah and you have to go and see blah, blah, blah?’ It was for his heart and that too, and I said ‘are you supposed to’, and he goes, ‘cause when I saw him he was like, ‘oh yeah, okay, oh I can do that later’. And I said, ‘look, that’s why I love it here because I can just get the car and take him straight into hospital’. (FG-1)

Earlier in the same focus group a participant reflected that they *‘feel like a free range chicken working here*’ because of the flexibility and support and the culture of building relationships and trust with street whānau.

One participant reported trying hard to *negotiate* with someone whom she was supporting, to see one of the Organisation’s on-site GPs about the severe pain he was experiencing:

*We tried for ages to get him to come to [on-site primary care clinic] for a walk-in clinic ‘cause he was saying ‘I’m in a lot of pain, I’m in a lot of pain’, but it was really hard to get him there. His timing is, we call it ‘[Name of person] time’. Like, he runs on his own time. Yeah, and I want to get from him ‘what sort of care do you want, what would appropriate medical care look like?’ But it’s a real challenge with him because he doesn’t buy in. It’s like he wants it but he doesn’t want, he wants it to appear on his doorstep rather than going out to seek it.* (FG-3)

Another participant mentioned that, even when they had managed to undertake the *doing* work of getting street whānau to the setting of their appointment, waiting times could present another barrier. Through this discussion the limits of what the housing key workers could achieve through *negotiation* were evident:

*Yeah, and they’re so influenced, peer influenced as well. So I’ve seen heaps of times someone waiting for their appointment which is coming up in, like, 20 minutes, but if Joe Bloggs walks past and goes, ‘hey bro, what are you up to? We’re just gonna go to this’., ‘Oh yeah, I’m coming’. I’m like, ‘Oh, you were so close, but I’m not here to force you to see the doctor. I want you to see the doctor, but you also do what you want to do’*. (FG-3)

### We need a bigger approach: the role of housing key workers in supporting healthcare interactions

Housing key workers played an important role in supporting street whānau when they did access healthcare. This included visits to the GP (typically at the Organisation’s on-site clinic), or visits to outpatient hospital appointments or to the emergency department, and hospital admissions. This work often started long before the time of the appointment and was rooted in the housing key worker’s *knowledge* about the street whānau and their wider context. This was evident in examples given of the ways in which housing key workers prepared street whānau as to what might happen when they met a healthcare professional. One participant said:

*I do a lot of prep work before I go anywhere, like, I will actually sit down with my whānau and pretend, like, this is what the doctor’s gonna ask you, or, this is what the psychiatrist is going to ask you. You know, so that they have an idea.* FG-4

It was also evident that housing key workers *knowing* street whānau was often critical to a successful healthcare visit, for example with regard to the use of appropriate de-escalation techniques if needed while waiting for the appointment and during the appointment itself.

On arrival at a hospital outpatient appointment, housing key workers often had to *negotiate* again, this time with reception staff before they would agree to even check whether street whānau had an appointment. Some receptionists were characterized as ‘gate keepers’ who appeared as if they would prefer ‘not to let them in’. This was discussed in a focus group where participants noted that it was common for street whānau to be told they didn’t have an appointment, without the receptionist ‘even looking on the screen’. This discrimination towards homeless people was also evident in descriptions of some healthcare staff’s attitudes and behaviours towards them, something that all of our participants found very distressing:

*I feel like the words are too deep, and I might become too [emotional]. Like, ‘you’re trash’, or ‘you’re homeless. You’re a homeless drug addict with mental health issues, you’ve got no choice but to take what we’re giving you and we’re not gonna give you any other options that might be better for you because this option that I’m giving you is the easiest’. And that’s really evident, it’s not like a one-off experience. I would say I’ve had that experience nine out of the ten times I’ve gone with someone to access medical care or support.* (FG-3)

The difficulties of unfamiliar and potentially re-traumatising healthcare spaces for street whānau were also discussed. This was evident in the following story a participant shared:

*It’s this bio-medical response, you know? It’s a western response that just doesn’t fit our people, homeless people. It’s just very clinical and there’s no relationship. We have one at the moment … she’s got a low blood count and iron, something that’s so easily treated, but the amount of time and resources that’s gone in to try and get this young woman to hospital, because she’s really agitated, she gets really abusing, she’s under treatment of mental health, she’s got extensive trauma. So the guards that are up for her are just huge. I just think staff aren’t trained well in how, and I don’t think they have enough time, because she literally needs someone to hand-hold her for a day. So three times they’ve tried to give her the procedure and she’s kicked off. Security have escorted her out of the hospital…So very simple procedures and the outcome could be really devastating for her aye?* (FG3).

This experience of retraumatisation often began in reception areas where housing key workers discussed having to *negotiate* to support individuals to wait to be seen. This was because they often became agitated because of the environment, not having anything to do to pass the time, and the reaction of other waiting patients to their appearance and smell – tangible signs of their homelessness. In this situation, housing key workers often had to deploy de-escalation techniques, as well as advocate for whānau.

This de-escalation and advocacy work continued into interactions with healthcare professionals. The example below highlights the complexity of the work required to enable street whānau to successfully complete a healthcare visit and demonstrates all aspects of care as *knowing*, *doing* and *negotiating*. Of note, this healthcare interaction occurred with the primary care clinic situated on the Organisation’s site with whom housing key workers already had a relationship, which no doubt contributed to the ability of the housing key worker to negotiate with the GP around the best way to work with this individual:

*I had a guy…whose got the really major distrust of services and it’s really hard to get him to [the primary care clinic]. He went [there] one day and then he called me, he’s like, ‘I’ve just had an argument with the doctor’, and I’m like, ‘do you need me to come down’? And I did. So he was wanting pain medication for his ribs, which had been broken. The doctors were, ‘no, we can’t give it to you, we can only give you this small amount because your ribs should be fine based on how long it’s been’. And he just got very, like, agitated and left, like, feeling like he’s been classed as a druggie, this, that or the other. So I came down and I talked to the doctor, like, ‘this is why he’s getting quite agitated. He’s got a mistrust of doctors, he feels like he’s been treated as a druggie, blah blah’. And the doctor’s like, ‘oh okay, I understand that, thanks for explaining it’. And then they said, you know, ‘we’ll give you one week’s worth or two-week’s worth as a low dose’.* (FG-3)

However, not all healthcare staff were receptive to the information shared by housing key workers. The attitudes and behaviours of healthcare staff often prevented housing key workers in deploying the strategies they knew would keep street whānau calm while they were being treated, particularly in hospital settings.They spoke about how hospital clinicians often ignored the advice of housing key workers, for example around the need for street whānau to have an alcohol plan put in place or to have a translator present. Healthcare staff frequently did not recognise the housing key worker’s expertise, particularly their ability to work in a trauma-informed way, or to draw on their in-depth *knowledge* of their street whānau. As a result, participants discussed how healthcare encounters with mainstream hospital services could end up with whānau being re-traumatised, as healthcare staff often did not seem to understand the impact of previous and current trauma on physical and mental health. One participant stated:

*One of the big barriers I see in the secondary services, is that they’re not aware of the effects of trauma on people’s health. I think physiologically as well as mental, I mean they’re both the same aren’t they, but you know, things like wound healing has such a significant effect of trauma. You know, you’re not going to heal as well if you’ve had a trauma history. You’re not going to behave the same as other people when you’ve had a trauma history. And every time we go to an ED, we typically retraumatise people, because they’re not aware of anything else other than putting the band aid on, yeah.* (FG-4)

Housing key workers also talked about their credentials being questioned, sometimes in an aggressive way, when they advocated on behalf of street whānau. For example, one participant shared being “backed up against a wall by a charge nurse… she wanted to know where I was from, who I was, and she literally was trying to intimidate me”. Participants also gave examples of being asked by hospital clinicians to leave the room during interactions because their support was misinterpreted as interfering, or because their de-escalation techniques, such as touching a shoulder, were seen as crossing a professional boundary. This created frustration as their intention was to keep both street whānau and health professionals safe.

Participants raised the power differential between themselves and medical doctors as a barrier to being taken seriously in a hospital setting. There was often a mismatch between the values guiding the practice of housing key workers and those underpinning medical practice. This was most clearly evident in discussions of autonomy and choice versus the risk of not following the medical advice given. One participant discussed an extreme case where this occurred:

*We had a guy with a fungating tumour…He was discriminated against, ‘cause he was passing blood, falling off dead skin and what-not. He was an alcoholic that wanted to keep his arm. They wanted to amputate. He had made a decision to keep the [arm]…and part of his reason was he wanted to remain on his cigarettes. And we tried and we tried and tried and we’re stuck, from a social work perspective, around autonomy, people’s choice. But I was just blown away because the hospital actually put out a warrant for his arrest, decided he didn’t have capacity because that was the choice he was making.* (FG-3)

Housing key workers found support for their work with street whānau from known hospital social workers with whom they had a good relationship. However, this service was not consistently resourced, and housing key workers recognized there was a need for improved interactions with hospital staff, particularly in relation to promoting good end-of-life care:

*I wish we could have a better connection with the hospital, like, you’ve got a good connection with the social worker with one young man. I’ve got various social workers that I have good relationships with and we have phone calls and we can sort things out. But we need a bigger approach to that so that when we do get the issues of end of life, we can do it really smoothly.* (FG-3)

### I’m so glad that I came in at her time, that she was familiar with me: the role of housing key workers in supporting dying

Even when a diagnosis of a life limiting condition had been made and, on rare occasions, specialist palliative care accessed and/or an Advance Care Plan made, street whānau engaged with these services in unique ways that still required on-going support from housing key workers. For example, one man, whom a housing key worker defined as a ‘chronic alcoholic’ with untreated Hepatitis C, accessed hospice for two weeks and when discharged was admitted to hospital-level care in an aged residential care facility. The housing key worker reported that he continued his usual activities: ‘he came into town every day and window-washed and drank alcohol’, but eventually was so unwell he needed hospital care. Unusually, this man had completed an Advance Care Plan in which he stated he did not want to die in hospital. Therefore, the housing key worker reported that they ‘drove him back to the rest home and he passed away that night’.

This story was rare amongst those told. More often housing key workers were left upset, and sometimes used language such as ‘traumatised’, to describe their reaction to the unjust dying circumstances of their street whānau. One poignantly talked of a ‘profound sadness’ pervading the work they do because of all the ‘lives cut short’ they witnessed. Nevertheless, participants also talked about it being a ‘privilege’ and feeling ‘proud’ to support individuals through serious illness and dying. It was evident through the many examples provided that the *knowing, doing* and *negotiating* work they undertook to support their whānau continued up until the time they died. As one participant shared:

*I had to negotiate with her in the hospital. So it was quite amazing on the last day, because in the hospital I was, you know, she was kicking all the doctors and nurses out. And so I’m glad that I came in at her time, that she was familiar with me. And then she was listening to my kōrero (conversation), and I was firm, and I was saying, ‘okay, you listen to the doctors. Let them do what they need to do, and we’re going to get you in this place, okay? And I’m just going to go and inquire about it’. And it wasn’t, I think it was either that day or the next day that she passed. Yeah, so I kept her up to date with current information in regards to what her goal was. And that was, she just, she didn’t want to go into palliative care.* (Int-5)

## Discussion

This paper confirms the central role played by housing key workers in supporting healthcare interactions for people experiencing homelessness and vulnerable housing, including during dying. Our housing key worker participants often described their relationships with their clients as family-like (see also [15]). They felt their organisational culture supported their focus on relationships and awareness of the context for behaviours. This was evident in descriptions of the support they provide in relation to different stages of healthcare access, from recognising a health problem is present and healthcare required, to attending healthcare appointments and experiencing hospital admissions. As in our work with family caregivers [28], it was clear that the *relationship* between the housing key worker and their street whānau was central to supporting healthcare access and interaction. Drawing on the *knowing*, *doing* and *negotiating* framework developed from our caregiver work [28] enabled recognition of both the complexity, and expert nature, of the work they undertake to support their street whānau to access the healthcare they needed. Doing involved *knowing* street whānau in order to understand what would support them to access healthcare (and knowing how healthcare works), *negotiating* with them (and with health professionals) as to how this could be achieved, and *doing* the work of accompanying them to appointments and telehealth interactions. Below we consider the implications of these findings for the provision of palliative care to people experiencing homelessness and vulnerable housing who are currently underserved by palliative care [9].

Firstly, the barriers we identified to healthcare access for this group, and their low use of health services in relation to need, limit opportunities for specialist palliative care referral and/or adoption of a palliative approach to care by non-specialists. The nature of these barriers is in line with previous evidence from other countries [15, 30] and confirms that healthcare environments are not safe spaces for people experiencing homelessness [11]. Participants discussed how previous negative and traumatic experiences of institutions, and some of those that work within them, lead many people experiencing homelessness to avoid them even when they understand that death is a potential outcome of not seeking care [31]. This mistrust is compounded by competing demands on their time, the use of alcohol and other substances, and the impact of serious mental health problems [15]. At a structural level, health service contact relies on access to phones and a fixed address. For Māori and Pacific people there is the known overlaying challenge of the on-going trauma of colonisation, institutional racism, and the lack of culturally safe healthcare [32]. Finally, participants discussed the social death [33] many of their street whānau felt they had already experienced, which led them to feel as though their death would not be something anyone cared about, even if they did engage with health services. Overall, it was evident that the ability and even potential to provide palliative care for street whānau depended upon supporting engagement with mainstream health services. These interactions also offered opportunities for identifying the need for palliative care, as referral to hospice for street whānau was reported to be rare.

Secondly, the relationship between the housing key worker and person experiencing homelessness or precarious/vulnerable housing had developed over a long period of time with a high degree of skill, and was underpinned by strong values. When such relationships work well, they involve key workers being receptive and responsive to the priorities of street whānau. This is critical because health is rarely perceived by street whānau as the main priority. Developing a relationship of this nature would be difficult, if not impossible, for those working within either generalist or specialist palliative care, given limitations of time and the range of non-healthcare related needs among the homeless community [34]. As such, our findings strongly support the recommendation of Stajduhar et al. [15] that a systems-level focus on developing working partnerships between healthcare professionals and housing key workers is crucial to developing new models of palliative care for people experiencing homelessness and precarious/vulnerable housing.

An important component of developing such relationships is shifting perceptions of what palliative care means within the context of homelessness and precarious/vulnerable housing, both for healthcare professionals and for housing key workers. Upskilling social care providers, including housing key workers, in palliative care has been recommended elsewhere [15, 35] and recent initiatives have shown some success [36]. A UK study found that a two-day training course for workers at a hostel for people experiencing homelessness was useful in terms of improving knowledge and confidence relating to palliative care. The authors concluded there was a need to embed such training within routine practice [35]. Similarly, creating opportunities to upskill generalist palliative care providers on the nature of dying within the homeless community, as well as on the skills and expertise housing key workers possess, could sensitise practitioners to considering alternative models of care than those currently on offer to this community. Training in trauma-informed care should also be prioritised, and critically, should extend to non-clinical people in emergency, outpatient, and palliative care health settings including receptionists and security personnel.

However, education and training alone cannot address the existing power imbalance between housing key workers and health care professionals within mainstream health settings (see Table 2 in Appendix). Indeed, despite being in roles that often already have long established trusted relationships with the people they work with, housing key worker professionals can be viewed as lacking the necessary health knowledge required to support vulnerable populations. This finding is in line with recent research exploring health and social care professionals’ views of dying at home for people experiencing deprivation in Scotland found that social sector staff do not believe they are seen as equal partners by healthcare professionals, or that the expertise and knowledge they have relating to working with people experiencing structural disadvantage is recognised [34]. Partnership models developed to support working between generalist and specialist palliative care providers [37] may provide guidance as to how partnership can be achieved in practice through reciprocal learning and recognition of distinct, but complementary, skill sets. However, this approach requires a commitment to genuine partnership and respect between service providers in which they acknowledge each other’s unique skills and knowledge. In doing so, the reciprocity of shared learning can be enhanced. Moreover, integrating someone with skills in palliative and end of life care into a team of housing key workers may indeed be more effective than providing an ad-hoc, referral dependent in-reach service from an external service such as hospice [19].

## Limitations

This paper does not include the perspectives of people with lived experience of homelessness. Whilst we conducted interviews with street whānau as part of this study and these are given appropriate focus in a separate paper, it is clear key workers were fundamental supporters of many street whānau. Their views provide a unique and mostly unexplored perspective critical to the application of palliative care for this population. Ethnographic methods may have enabled a more in-depth insight into the work of housing key workers, but were beyond the resources of the project.

## Conclusion

Whilst the growing commitment to centre equity in palliative care is good to see, how this can be translated into tangible improvements in end-of-life experiences for communities experiencing structural inequities is unclear. Our findings support the need for new models of palliative and end of life care for people experiencing homelessness which recognise the central role played by housing key workers in supporting healthcare access. There is also a need to identify strategies to support the recognition and introduction of a palliative approach to care, or specialist palliative care referral, within mainstream, tertiary hospital settings.

## Supplementary Information

Supplementary Material 1.

## Data Availability

The dataset generated during the current study is not publicly available as this would compromise participant anonymity but is available from the corresponding author on reasonable request.

## Appendix

**Table 2:** Recommendations for palliative care services and health professionals wanting to develop new models of palliative care for people experiencing homelessness

Principle	Recommendation
Secure housing is directly linked to health and well being at the end of life	Establish relationships with people in housing key worker roles and the organisations supporting people experiencing homelessness.
Housing key workers have unique knowledge and expertise	Recognise the expertise held by people in these roles. They may be the ‘significant other’ and primary support for the person experiencing homelessness. They are often one of the only support person available – listen to what they have to say.
Being homeless can be life limiting for most people	Be prepared to accept referrals that do not appear to meet the typical diagnostic or prognostic palliative care criteria. Be prepared as death can often appear sudden.
Recognise opportunities to integrate a palliative care approach	All health care professionals need to recognise the value of integrating a palliative approach to care, particularly within emergency departments which are critical points for people experiencing homelessness to receive health care. Housing key workers have a role in recognising and advocating for this approach to care when having contact with mainstream health services. Their support in interactions between professionals and people experiencing homelessness also provide opportunities for identifying the need for palliative care.
Palliative and end of life care is complex for people experiencing homelessness	Complexity is impacted by a social context that includes mental health, addictions and lifelong trauma, not just complexity of medical condition. This will impact on management of total pain, including financial, psychological and social elements [38].
Inflexible mainstream health systems can cause more trauma	Flexibility in service provision is essential [39]. For example, people who need to be supported when they cannot keep to appointment times/keep in touch in regular ways, eg, do not have a phone or regular postal address. If people do not attend appointments, try and find out why.
Mainstream healthcare, including hospice is not readily accessed	Healthcare settings are often not perceived as safe spaces. Working with people with trusted relationships with people experiencing homelessness is crucial. There is potential to develop reciprocal learning and relationship building models [37] to facilitate palliative care in this context.
Housing key workers are exposed to sudden and traumatic death and dying	Just as caregivers need support, so do people in these roles as they face a lot of (unjust) death and dying. Offer ongoing training, upskilling, and support, including during bereavement.
Training for trauma-informed care needs to be prioritised	Training needs to include non-clinical personnel (eg, receptionists, security) working in health care settings such as primary care, emergency and outpatient care, and palliative care settings, who play a crucial role in enabling or preventing potential patients from accessing care.
