# Supplementary material for: The central role of housing key workers in supporting healthcare interactions for people experiencing homelessness and implications for palliative care: a qualitative study
Source: BMC Palliat Care. 2024 Dec 2;23:275. doi: 10.1186/s12904-024-01598-x (PMC11613513; doi:10.1186/s12904-024-01598-x)
Supplement: Supplementary file 1 — Supplementary Material 1. [file 12904_2024_1598_MOESM1_ESM.pdf]

**Appendix 1: Focus group/interview schedule**

*Introduce yourself and provide time for questions they may have about the study.*

*Confirm that you have received written consent from all those present.*

*Explore both positive and negative experiences.*

**Staff experiences of providing care and support**

In your role as a provider of health and social care at [this organization], tell us about your experiences of providing care and support for people with a serious life limiting illness?

In your experience, what impact do you think being homeless has on people's ability to access in health and social services in the context of having a serious life limiting illness?

**Impact of deprivation and homelessness**

What are the impacts of being homeless for people living with a serious life limiting illness as they near the end of their lives?

**Strengths of a community**

What support do people generally get from family and friends? What does that support consist of?

What support do people generally receive from within the community? What does that support consist of? What do you think that support means to them?

What are the strengths of the homeless community in supporting people with a serious life limiting illness? What are the challenges?

**Research strategies**

What do you this are the most important research priorities for your population?

What are the best ways to connect with the community in order to understand their experiences? Challenges and opportunities?
